# Atropia in Traumatic Tetanus

**Published:** 1880-02

**Authors:** 


### ATROPIA IN TRAUMATIC TETANUS.

Surgeon D. H. Cullimore, F. R. C. S. L., &c., in the *London Lancet* for December, gives the following deductions from a case of Traumatic Tetanus under his observations at Rangoon, Burmah, in April, 1875, which yielded to the subcutaneous administration of atropia in $\frac{1}{40}$ to $\frac{1}{60}$ grain doses, every 5 to 8 hours.

1. That tetanus, which is a series of reflex phenomena, depending upon an over-excited or congested state of the brain, the spinal cord and their membranes, is capable of being relieved or even cured by atropia, when administered in comparatively small doses, extended over a certain period of time, according to the severity of the symptoms, though we know from the experience and experiments of Drs. Harley, Frazer and others, that when given to its full physiological effect, it produces excitement and congestion of the cord, followed by the usual reflex results, as jactitation, muscular spasm and convulsive fits.

2. That the administration of the medicine was not followed by any of the easily recognizable symptoms of the drug (two grains of which has caused the death of a healthy adult when given in one dose) proving both the tolerance induced by the disease, and, perhaps, also illustrating the homœopathic theory or formula *sine* the infinitesimal system of dosage.

3. That amputation of the injured part, recommended so strongly by Larrey and others, even after the supervention of tetanus, though it may perhaps help to lessen the severity of the disease, does not act as a prophylactic, and should, I think, never be had recourse to after the symptoms have declared themselves. It would be then injurious, for the peripheral irritation would have become central and independently dynamic. For the same reason the division of nerves should not be resorted to. In two cases where I examined the nerves after death, I failed to perceive that they differed in any way from those of the opposite side. In one of these there was slight congestion of the membranes and a softening of the cord in the lumbar region, and in the other, a peculiar cloudiness of the cord, which may, however, have been due to post mortem changes. Yet it is certain that there is some lesion, though in every case, we may not be able to perceive it. This lesion should be looked for in that portion of the spinal cord with which the nerves from the affected part first communicate.

4. If the line of treatment adopted in this case should be found beneficial in others of the same disease, I would suggest that it might be extended, with such modifications as may be necessary to the treatment of allied diseases, as epilepsy, puerperal convulsions, and hydrophobia.

